# Skin Cancers in People Living with Human Immunodeficiency Virus (HIV) Infection

**DOI:** 10.3390/jcm14186447

**Published:** 2025-09-12

**Authors:** Giulia Ciccarese, Liberato Roberto Cecchino, Fedele Lembo, Sergio Ferrara, Chiara Grillo, Cristina Pizzulli, Piergiorgio Di Tullio, Paolo Romita, Caterina Foti, Francesca Sanguedolce, Domenico Parisi, Francesco Drago, Aurelio Portincasa, Sergio Lo Caputo

**Affiliations:** 1 Dermatology Unit, Department of Medical and Surgical Sciences, University of Foggia, Viale L. Pinto, 1, 71122 Foggia, Italy; 2 Unit of Reconstructive and Plastic Surgery, Department of Clinical and Experimental Medicine, Ospedali Riuniti di Foggia and University of Foggia, Viale L. Pinto, 1, 71122 Foggia, Italy; liberato.cecchino@unifg.it (L.R.C.); fedele.lembo@unifg.it (F.L.); domenico.parisi@unifg.it (D.P.); aurelio.portincasa@unifg.it (A.P.); 3 Infectious Diseases Unit, Department of Medical and Surgical Sciences, University of Foggia, Viale L. Pinto, 1, 71122 Foggia, Italy; sergio.ferrara@ospedaliriunitifoggia.it (S.F.); chiaragrillo.infettivi@gmail.com (C.G.); sergio.locaputo@unifg.it (S.L.C.); 4 Unit of Pathology, Department of Clinical and Experimental Medicine, University of Foggia—University Hospital “Policlinico Riuniti”, Viale L. Pinto, 71122 Foggia, Italy; cristina.pizzulli@unifg.it (C.P.); francesca.sanguedolce@unifg.it (F.S.); 5 Unit of Medical Oncology and Biomolecular Therapy, Department of Medical and Surgical Sciences, University of Foggia, Viale L. Pinto, 1, 71122 Foggia, Italy; pgfg@libero.it; 6 Section of Dermatology, Department of Precision and Regenerative Medicine and Jonian Area, University Aldo Moro of Bari, Piazza G. Cesare, 11, 70124 Bari, Italy; paolo.romita@uniba.it (P.R.); caterina.foti@uniba.it (C.F.); 7 Section of Dermatology, Casa di cura Villa Montallegro, Via Monte Zovetto, 16145 Genoa, Italy; francescodrago007@gmail.com

**Keywords:** human immunodeficiency virus infection (HIV), skin cancer, skin malignancy, people living with HIV

## Abstract

**Background/Objectives:** The advent of combination antiretroviral therapy has led to significant reductions in HIV-related morbidity and mortality and, conversely, an increasing incidence of chronic diseases, such as cancer. This study aimed to assess the incidence of skin malignancies in a cohort of people living with HIV (PLWH) compared to HIV-uninfected individuals (HUPs). **Methods**: Between April 2023 and April 2025, PLWH attending the Infectious Disease Unit at Policlinico of Foggia, Italy, were invited for skin cancer screening (cases). During the same period, patients visiting the Dermatology Unit were asked to undergo skin cancer screening and a rapid HIV test. Those who tested negative were included as controls. Suspicious lesions were surgically excised at the Plastic Surgery University Unit and examined by a dermatopathologist. **Results:** We enrolled 91 cases and 91 controls. Precancerous and cancerous skin lesions were detected at similar rates in PLWH and HUPs (12% vs. 13.2% and 7.6% vs. 8.7%). The total number of cancerous and precancerous lesions was higher in the PLWH group. In both groups, basal cell carcinoma was the most common tumor. Squamous cell carcinoma, basosquamous carcinoma, and dermatofibrosarcoma protuberans were found only in PLWH. **Conclusions**: The higher risk of multiple and rare skin cancers in PLWH should be recognized by healthcare providers and patients. PLWH should have regular skin cancer screenings, especially if they have additional risk factors such as a history of extensive ultraviolet radiation exposure.

## 1. Introduction

### 1.1. Pathogenic Mechanisms of Immunosuppression

Immunosuppression can result from primary inherited disorders [1] or secondary causes, such as diseases or medications [2]. Common causes of immunosuppression include lymphoproliferative disorders (primarily non-Hodgkin lymphoma and chronic lymphocytic leukemia), human immunodeficiency virus (HIV) infection, acquired immunodeficiency syndrome (AIDS), and the use of immunosuppressive treatments for various conditions, such as autoimmune diseases and solid organ transplantation [2,3,4,5].

Both branches of the immune system, innate and adaptive, are responsible for identifying and removing neoplastic cells and microbial agents. As part of the innate immune system, macrophages and natural killer (NK) cells can phagocytize cellular debris, cancer cells, and microbes. More specifically, NK cells bind to cancer cells and virus-infected cells, which are characterized by the loss or alteration of their self-markers, resulting in the presentation of these altered self-markers to the major histocompatibility complexes (MHCs) [6,7]. Through the release of cytotoxic granules into target cells, NK cells can induce cell death. Cytotoxic T lymphocytes (CTLs) are a key component of the adaptive immune system and play a crucial role in destroying cancer cells. CTLs, primarily T CD8+ cells, recognize tumor cells by detecting tumor-associated antigens presented on MHC class I molecules. Once activated, CTLs initiate two main pathways related to apoptosis: the perforin-granzyme pathway and the Fas-Fas ligand interaction. In the first pathway, CTLs release perforin, which forms pores in the target cell membrane, allowing granzymes to enter and trigger apoptosis. Additionally, CTLs express Fas ligand, which binds to the Fas receptor on the tumor cell, activating a signaling cascade that leads to apoptosis [6,7]. All these mechanisms are essential for immune surveillance and the elimination of neoplastic and virus-infected cells [6]. Regardless of its origin, a weakened immune system increases the risk of malignancies and infections compared to the general population [2,3,4,5,8].

### 1.2. Drug-Induced Immunosuppression

Regarding the iatrogenic causes of immunosuppression, immunosuppressive drugs work by depleting T lymphocytes, which is essential in organ transplant recipients (OTRs) to lower acute rejection rates and improve graft survival. At the same time, these drugs reduce immune surveillance, allowing abnormal cells to survive and grow. Among OTRs, the risk of cancer is 2 to 5 times higher compared to the general population, with skin cancers being the most common malignancies in these patients [5,9,10,11].

In addition, several anti-cancer treatments have immunosuppressive effects and may cause, among their adverse events, skin cancers. The increased risk of skin cancers is linked to classic immunosuppressants such as methotrexate (MTX), chemotherapeutic agents like fludarabine and hydroxyurea (HU), targeted therapies such as ibrutinib and Janus Kinase inhibitors (JAKi), mitogen-activated protein kinase pathway (MAPKP) inhibitors, sonic hedgehog pathway (SHHP) inhibitors, and radiotherapy. The oncogenic mechanisms are diverse [12]. For example, MTX has immunosuppressive and photosensitizing effects; mammalian target of rapamycin (mTOR) inhibitors suppress the immune system and may cause mutagenesis in normal tissues; HU inhibits DNA synthesis and repair in skin cells, leading to mutations, and ibrutinib increases photosensitivity [12].

### 1.3. Ultraviolet-Induced Immunosuppression

Beyond medications, ultraviolet radiation (UV) is a key environmental risk factor for skin cancer in OTR. UV damage results from the production of reactive oxygen species (ROS), causing ‘oxidative stress,’ which is an imbalance between these highly reactive molecules and the body’s ability to detoxify them. ROS production leads to skin inflammation and immunosuppression, processes that promote skin cancer either independently or through their connection. These events affect more than just the skin, as UV-damaged biomolecules can enter the bloodstream, causing systemic oxidative stress and inflammation [13]. Additionally, UV radiation may induce skin immunosuppression by depleting Langerhans cells and decreasing epidermal antigen-presenting cell function, which increases the skin’s vulnerability to carcinogenic stimuli [13].

### 1.4. HIV-Induced Immunosuppression

Regarding infectious conditions that lead to immunosuppression, HIV infection is the primary cause. Before significant depletion of CD4+ T cells, HIV can cause impairments in various CD4+ T cell functions, including T-cell colony formation, autologous mixed lymphocyte reactions, expression of interleukin-2 (IL-2) receptors, and IL-2 production [14,15]. Additionally, T cells’ proliferative responses to several stimuli are diminished throughout all stages of HIV disease [14], and HIV may also contribute to cancer development through a direct oncogenic effect, mainly via the tat gene [15,16].

Beyond its known impact on quality of life, mood, and sexual function of the affected patients, as described in other chronic STIs [17], HIV infection also causes an increased risk of developing cancers [18].

### 1.5. The Risk of Cancers in People Living with HIV (PLWH)

Traditionally, malignancies in people living with HIV (PLWH) are classified as AIDS-defining and non-AIDS-defining cancers [19]. AIDS-defining cancer is considered a type of cancer that PLWH are at high risk of developing; indeed, the presence of one of these cancers in PLWH strongly suggests AIDS [19,20,21]. AIDS-defining cancers include Kaposi sarcoma, non-Hodgkin lymphoma, and cervical cancer [19,20,21].

Except for these three tumors, all other cancers occurring in PLWH are considered non-AIDS-defining and include HIV-associated tumors and incidental cancers. HIV-associated cancers are types of cancers that are more likely to occur in PLWH than in HIV-uninfected people (HUPs): Hodgkin lymphoma and cancers of the liver, lung, mouth, throat, and anus. Beyond HIV infection, other factors such as older age, heavy alcohol or tobacco use, and infection with other viruses—such as human papillomavirus (HPV), Epstein–Barr virus (EBV), and hepatitis B or C viruses—may increase the risk of developing these tumors [22,23,24,25,26]. Indeed, coinfection with HIV and at least one of these oncogenic viruses may result in malignancies due to diminished immune surveillance against viruses and virus-infected tumor cells [26]. HIV infection also increases the risk of other cancers, including myelodysplastic syndrome, polycythemia vera, and various skin cancers such as squamous cell carcinoma and Merkel cell carcinoma [18,24,27].

The advent of combination antiretroviral therapy (cART), which includes several drugs from different antiretroviral classes, since the mid-1990s, marked a major turning point in HIV/AIDS treatment, leading to significant reductions in HIV-related illness and death. cART is defined as two or more nucleoside reverse transcriptase inhibitors (NRTIs) combined with at least one protease inhibitor, one non-nucleoside reverse transcriptase inhibitor (NNRTI), or an abacavir-containing regimen of three NRTIs [21,25,26,27,28]. The widespread use of cART has resulted in a substantial decline in opportunistic infections among people living with HIV (PLWH), greatly improving life expectancy to levels similar to those of the general population. As a result, the aging of PLWH has become more common, bringing about a rise in other chronic diseases, such as cancer. Thanks to cART, the epidemiology of cancer has shifted, with a decrease in AIDS-defining cancers and an increase in non-AIDS-defining cancers [29,30,31]. Consequently, a growing proportion of cancers—such as colon, breast, and prostate—are now common incidental cancers that may affect PLWH [19].

Regarding skin cancer incidence in PLWH, especially after the advent of cART, conflicting data are present in the literature [29,30,31]. Additionally, no standardized guidelines exist regarding the value of periodic skin cancer screening in this population.

Therefore, the rationale of the present study was to evaluate the risk of skin cancer in a specific category of immunosuppressed subjects, specifically those with PLWH.

More specifically, the present study aimed to assess the incidence of cutaneous malignancies in a cohort of PLWH accessing a tertiary hospital in southern Italy (the case group) compared with the incidence of skin cancers in the general population (the control group). Secondary aims included evaluating the potential association between these cancers and immunological parameters, as well as HIV RNA load in plasma and CD4+ T cell count in blood.

## 2. Materials and Methods

This study is based on a retrospective analysis of a prospectively collected database. Between 1 April 2023, and 1 April 2025, patients aged 18 years or older who consecutively visited the HIV outpatient clinic of the Infectious Disease Unit at the Policlinico of Foggia, Italy, were invited for a dermatologic visit to screen for skin cancer. Both patients with a new, recent HIV diagnosis and those with an existing HIV infection undergoing cART treatment were included (cases). A dermatologic examination was performed on the same day as the routine HIV visit at the Dermatology Unit of the same hospital. A trained dermatologist assessed the presence of skin lesions through a thorough, full-body examination supported by a handheld dermoscope (DL5 dermoscope [DermLite, Aliso Viejo, CA, USA]).

During the same study period, patients visiting the Dermatology Unit for any reason were invited to undergo a comprehensive full-body skin examination for cancer screening by the same dermatologist. They were also offered a rapid HIV screening test after signing a written informed consent form. The test used was the DetermineTM HIV Early Detect test (Abbott Rapid Diagnostics S.r.l., Sesto San Giovanni, Milano, Italy), which is the first and only World Health Organization (WHO)-prequalified fourth-generation rapid diagnostic test. It detects the HIV-1 p24 antigen using finger-prick blood samples with a sensitivity of 95%. The wait time for results was 20 min [32]. Patients with a negative rapid HIV test (HUPs) result participated in the study as controls.

All suspicious skin lesions, in both groups (cases and controls), were surgically excised in the Plastic Surgery University Unit (Policlinico of Foggia, Foggia, Italy) and evaluated by a trained dermatopathologist of the same Hospital. A dermatologic consultation has been performed after the delivery of the histological results of the surgically excised lesions.

All patients who tested positive for skin cancers were discussed in the interdisciplinary team to plan any further surgical approach (when indicated), due to the aggressive behavior of some of these skin cancers [33]. This protocol is mandatory to reduce the need for more invasive surgery and complex reconstruction, the risk of complications, recurrence and lymph node metastasis [33].

Demographic and clinical data, including the intake of photosensitizing drugs, as well as histopathologic analysis, were collected for both patient groups in an Excel database.

For PLWH, the clinical history of HIV infection and laboratory data (CD4+ T cell blood count and HIV RNA load in plasma) were also collected. From both clinical and laboratory perspectives, the definition of an undetectable viral load has evolved. Today, it is most commonly understood as an HIV RNA viral load in plasma of <20 copies/mL, measured by the nucleic acid amplification technology PCR [34]. In this study, HIV RNA load in the plasma of PLWH was calculated using the Abbott Real-time HIV-1 Amplification Reagent Kit (Abbott Molecular Inc., Des Plaines, IL 60018, USA).

Categorical variables were presented as absolute numbers and percentages, while continuous variables were shown as means and standard deviations (SD).

The characteristics of patients with and without HIV infection were compared using a chi-square test for categorical variables to evaluate potential associations with skin cancers. Categorical variables were compared with Fisher’s exact test, and continuous variables with the Mann–Whitney U test. The primary outcome results were reported as odds ratios (OR) with 95% confidence intervals (CI). A *p*-value of less than 0.05 was considered statistically significant. The analysis was conducted using R 6.2-0 software (The R Foundation for Statistical Computing, Vienna, Austria).

The study was conducted following the Declaration of Helsinki and was approved by the Institutional Review Board of OSPEDALI RIUNITI DI FOGGIA (protocol code 104/C.E./2020, dated 30 September 2020).

## 3. Results

During the study period, 91 PLWH (cases) and 91 HUPs (controls) were recruited. Among the cases, most patients were males (78.8%) with a mean age of 52.3 years (±12.0, range 30–80); among the controls, both sexes were equally represented (49.4% males and 50.6% females) with a mean age similar to that of the cases (49.9 years, ±17.8, range 19–84). In both groups, the majority of patients were over 50 years old. The most common phototypes were II and III; however, among the cases, six patients (6.5%) had a dark phototype (IV–VI) compared to only one control (1.1%). A melanoma family history was found in a minority of patients, while signs of photodamage (pigmented spots, wrinkles) were frequently observed in both groups (49.4% of cases and 52.7% of controls). The use of photosensitizing drugs for more than 5 years and immunosuppressive drugs, both of which can increase skin cancer risk, was recorded equally in cases and controls. The total body nevus count was fewer than 50 in most subjects (84.6% of cases and 86.8% of controls).

No statistically significant differences were found in the demographic, clinical and histological features between PLWH and HUPs (*p* > 0.05), except for the gender: the male sex was most represented among the cases than the controls (*p* = 0.000061). Table 1 describes and compares the demographic and clinical features of the patients recruited in the two groups.

Precancerous skin conditions, such as actinic keratoses and epithelial dysplasia, were identified at similar rates in cases (11/91 (12%)) and controls (12/91 (13.2%)). The diagnosis of actinic keratoses was made clinically with dermoscopy assistance [35], while the diagnosis of epithelial dysplasia was confirmed histologically after surgically removing a suspicious lesion.

The detection rate of skin cancers was similar between the two groups, as well (7/91 PLWH (7.6%) versus 8/91 HUPs (8.7%), OR 0.86, 0.29–2.49) (Table 2).

When focusing attention solely on PLWH, the diagnosis of the infection had been known for an average of 15.5 years (range 0–44 years), and most patients (89/91) were regularly taking cART. Only 2 of the 91 PLWH did not take cART: one patient because his HIV diagnosis had just been established; the other declined to take cART due to personal concerns about potential adverse effects of the medications. Both men were in the AIDS stage at the time of the dermatologic visit (CD4+ T cell < 200 cells/mm^3^) and received a histological diagnosis of KS, as expected [19].

The average number of CD4+ T lymphocytes in the blood of PLWH was 748.02 cells/mm^3^ (SD 412.15), and the average HIV RNA load in plasma was 16,630 copies/mL (SD 37,900.26). Excluding the two patients in the AIDS stage, the average CD4+ T lymphocytes count was 759.36 (SD 409.92), and the HIV RNA load had a mean value of 894.37 copies/mL (SD 8285.52). The HIV RNA load was considered undetectable (<20 copies/mL) in 79 out of 89 patients.

Among the PLWH, we compared the features of those diagnosed with skin cancer to those who are not. PLWH with skin cancers were slightly older (average age 53.7 years) compared to PLWH without skin cancers (average age 48.0 years). However, no statistically significant differences were found between PLWH with and without skin cancers regarding sex, age over 50 years, phototype, melanoma family history, photodamage, use of photosensitizing and immunosuppressive drugs, and number of nevi. Considering the CD4+ T cell blood count and HIV viral load in plasma, a CD4+ T cell count < 350 cells/μL and an HIV load > 20 copies/mL were significantly associated with skin cancers (respectively, *p* = 0.019362 and *p* = 0.015742). However, after applying the Yates correction to adjust the chi-square test, no statistically significant difference in skin cancer rates was observed.

We then analyzed the subjects who developed skin cancers to assess potential differences in tumor number, type, and location between PLWH and HUPs.

Analyzing only the subjects with skin cancers (7/91 cases and 8/91 controls), we found that all were males and that the average age was lower in PLWH (53.7 years) compared to HUPs (68.1 years). The number of skin cancers detected in PLWH (11 skin cancers in 7 patients, averaging 1.5 tumors per patient) was higher than in HUPs (9 cancers in 8 patients, averaging 1.1 per patient) (Table 3).

The skin cancers in PLWH did not have a worse prognosis compared to HUPs; specifically, the two cutaneous melanomas in PLWH were both superficial spreading melanomas (one “in situ”—Breslow thickness 0—and the other with a Breslow thickness of 0.1 mm). The two melanomas in HUPs were also superficial spreading melanomas (one “in situ” and the other with a Breslow thickness of 0.4 mm). None of these four melanomas showed histological ulceration. Therefore, the two in situ melanomas were staged as Tis melanomas, while the other two were staged as T1a melanomas (American Joint Committee on Cancer [AJCC], 8th edition [36]). T1 melanomas (those with a Breslow thickness of ≤1.0 mm) have an excellent prognosis, with an overall 10-year mortality rate of approximately 4% [36,37].

**Figure 1:**
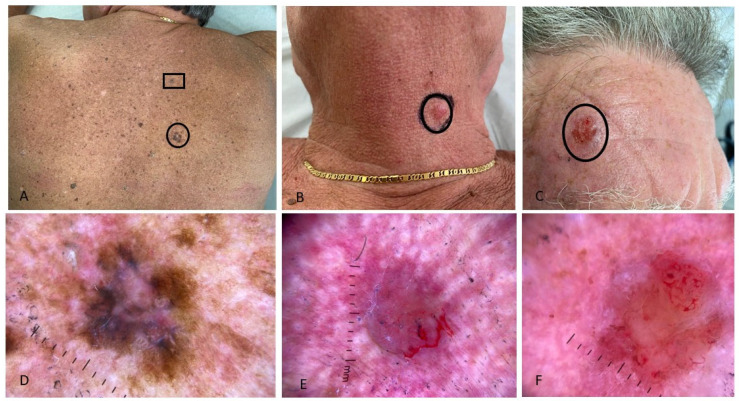
Multiple, concurrent skin cancers in a patient with HIV infection; (**A**) intense photodamage on the back; two pigmented flat lesions on the right shoulder histologically corresponding to two superficial spreading melanomas (one with Breslow thickness of 0.1 mm [black circle] and one “in situ” [black rectangle]); (**B**) pink nodule on the neck, histologically a nodular basal cell carcinoma; (**C**) erythematous, ulcerated nodule on the forehead, histologically a basosquamous carcinoma. Dermoscopy showed: (**D**) asymmetric pigmented lesion with irregular shape and variegated colors: the lesion’s periphery has an atypical pigment network, while the center shows a prominent bluish-white veil (referring to the lesion in the black circle in (**A**)); (**E**) pink-purple stroma, no pigment network, short fine telangiectasias, and focal ulceration at the lower edge of the nodule (referring to the lesion in (**B**)); (**F**) erythematous stroma, unfocused arborizing vessels, and white structureless areas (referring to the lesion in (**C**)).

**Figure 2:**
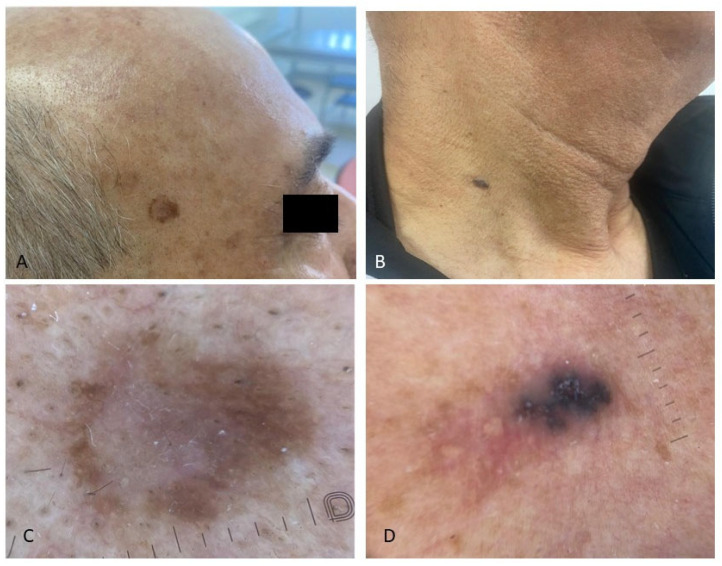
Simultaneous presence of precancerous skin lesions and basal cell carcinoma in the same patient living with HIV; (**A**) asymmetrical pigmented flat lesions on the right temple histologically corresponding to pigmented high-grade epithelial dysplasia, a precursor to squamous cell carcinoma; dermoscopy showed a white structureless area in the center (**C**); (**B**) bluish, nodular, oval lesion on the neck, characterized dermoscopically by blue-gray ovoid nests and milky-red areas (**D**).

**Figure 3:**
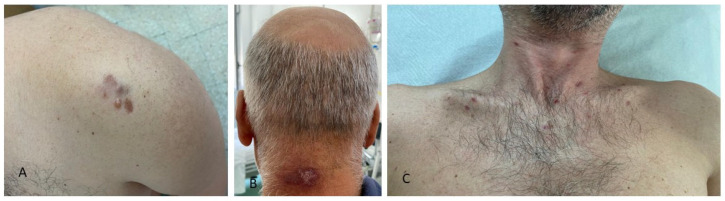
Clinical presentation of cutaneous malignancies in different patients living with HIV infection: (**A**) firm, red-to-violaceous plaques and nodules with hard consistency on the left shoulder, histologically corresponding to dermatofibrosarcoma protuberans; (**B**) purple-brown nodules with central hyperkeratosis on the neck; (**C**) violaceous papules and plaques on the chest, all histologically linked to Kaposi sarcoma in patients with AIDS.

In both groups, the most common skin cancer was basal cell carcinoma (BCC), accounting for 3 out of 11 cancers in PLWH and 6 out of 9 in HUPs. Cutaneous melanoma was equally represented among cases (2/11 cancers) and controls (2/9 cancers), while KS was more common in PLWH (2/11 cancers) compared to HUPs (1/9 cancers). Certain skin cancers, such as squamous cell carcinoma (SCC), baso-squamous carcinoma (BSC), and dermatofibrosarcoma protuberans (DFSP), were found only in PLWH. The most common site of skin cancers was the trunk in both groups (Table 3, Figure 1, Figure 2 and Figure 3). Patients signed informed consent forms for the publication of their clinical, histological data, and photographs.

Finally, we compared the number of patients diagnosed with precancerous skin lesions and the number of these lesions in both groups.

Precancerous skin lesions were found at similar rates in PLWH and HUPs (12% versus 13.2% and 7.6% versus 8.7%). Among the PLWH, 11 patients (10 males, 1 female; average age 65.6 years) were diagnosed with AKs and high-grade epithelial dysplasia; among the HUPs, 12 patients (8 males, 4 females; average age 65.0 years) were diagnosed with AKs.

The number of precancerous lesions observed in PLWH (50 lesions in 11 patients, averaging 4.5 lesions per patient) was higher than in HUPs (29 lesions in 12 patients, averaging 2.4 lesions per patient). In PLWH, the precancerous lesions consisted of 49 AKs and 1 high-grade epithelial dysplasia; in HUPs, the 29 precancerous skin lesions were all AKs.

## 4. Discussion

In our study, the demographic and clinical features of patients in both groups were similar regarding age, skin phototype, melanoma family history, photodamage, total number of nevi, use of immunosuppressive and photosensitizing drugs, and prior skin cancer diagnoses (*p* > 0.05). Concerning gender, the group of PLWH in our series showed a male predominance compared to the HUP group. Although more women worldwide live with HIV, in the World Health Organization (WHO) European Region, twice as many men are diagnosed with HIV each year as women [38,39].

The gender of PLWH in our series aligns with these data: in the group of cases, most patients were males (78.8%), compared with the control group (males: 49.4%).

Notably, we found a similar incidence rate of skin cancers in PLWH and in HUPs (7.6% versus 8.7%). The incidence rate of precancerous skin lesions was also similar (12% versus 13.2%). However, it should be emphasized that in our series, the PLWH diagnosed with skin cancers and/or cutaneous precancerous lesions were unaware of the risk associated with their lesions before the dermatologic consultation; therefore, these conditions would have remained undiagnosed indefinitely if they had not undergone a skin cancer screening. Conversely, HUPs had knowingly requested a dermatological consultation, in some cases with the specific aim of receiving a skin cancer screening.

Our study also highlighted the importance of regular cART use; indeed, all patients who did not take cART developed skin cancers (100%), as expected [19,21], compared to a small percentage of patients on cART (5/89, 5.6%).

The increased risk of skin cancers in immunosuppressed people compared to immunocompetent individuals is well established. However, most research on skin cancers in immunosuppressed individuals focuses on organ transplant recipients (OTRs). It is estimated that 30–70% of OTRs develop a skin malignancy, with squamous cell carcinomas (SCCs) being the most common, followed by basal cell carcinomas (BCC), Kaposi’s sarcoma (KS), Merkel cell carcinoma, and melanoma. Specifically, the risk of developing SCC in OTRs is 10- to 250-fold higher than in immunocompetent patients [3,5,9,10,11]. Due to this high risk, international guidelines for transplant patients recommend a full skin examination before transplantation and at least once a year afterward [40,41,42].

Unlike studies on OTRs, the medical literature on the risk of skin cancer in PLWH is limited, and the results of existing studies are often conflicting [29,30,43,44,45,46,47,48,49]. Additionally, there are no formal consensus guidelines for skin cancer screening in PLWH, especially after the introduction of cART [47].

Only a few studies have examined the risk of skin cancer in PLWH compared to a control group. A Danish nationwide cohort study found a twofold increased risk of BCC and a fivefold increased risk of SCC in PLWH compared with an age- and sex-matched background cohort (that was not necessarily tested for HIV). Low nadir CD4+ T-cell counts were associated with an increased risk of SCC [50]. Similar results were reported by an older study from California, which compared PLWH with a cohort of HIV-negative subjects [48]. A recent study linking data from HIV and cancer registries in 12 US states during the cART era (1996–2018) calculated the standardized incidence ratios for non-keratinocyte skin cancers (NKSC) (KS, melanoma, cutaneous lymphoma), comparing the incidence to the general population. The authors found that the risk for most NKSCs was similar to the general population, suggesting that PLWH without specific risk factors for NKSC may not require intensive skin surveillance [46].

Other studies have looked at the occurrence of skin cancers in people living with HIV (PLWH), but they did not include a control group. A US study with 4490 participants found that 5.7% of PLWH developed at least one skin cancer [44], while a more recent Italian study with 97 individuals reported a rate of 15.5% [30]. Our study’s cohort of PLWH had a rate of 7.6%, which falls between these two rates.

In our study, we collected data on both keratinocytic and non-keratinocytic skin cancers. Despite the small sample size, we agree that PLWH are at a higher risk of SCC compared to HUPs [45,48]. In our population, SCCs were only detected in PLWH and never in HUPs. BCC was the most common cutaneous malignancy in both groups, more frequently in the controls. Interestingly, among PLWH, the clinical and histological features of skin cancers were more diverse compared to those in HUPs. We identified six different types of malignancies in our PLWH group, including rare tumors like BSC and DFSP, whereas only three types of skin cancers—BCC, KS, and MM—were recorded in HUPs (Table 3). Notably, in our series, 2 out of 7 PLWH with skin cancers (28.5%) were diagnosed with multiple tumors simultaneously, aligning with another US study, which reported a lower rate (16%) [44]. The occurrence of multiple tumors in the same subject within our control group was less common (1 out of 8 subjects, 12.5%) than in our case group (28.5%).

According to other studies [30,49], our PLWH diagnosed with skin cancers were slightly older than PLWH without skin cancers (mean age difference: 5.7 years), whereas no differences were found regarding sex, photodamage, number of nevi, family history for melanoma, CD4+ T cell count, HIV RNA load and time in antiretroviral therapy.

Therefore, we can conclude that the skin cancers in our series of PLWH were not solely linked to immune status, unlike the population of OTRs, whose risk of skin cancers correlates with a lower CD4+ T cell count [44]. As previously hypothesized [30,44], skin cancers in PLWH, especially after starting cART, could be related to traditional risk factors such as aging, skin phototype, and chronic ultraviolet radiation (UVR) exposure, consistent with the general population. Indeed, as PLWH are living longer and experiencing fewer AIDS-related cancers, non-AIDS-related cancers have become the most common tumors in this group [44]. Additionally, in our region, which overlooks the sea and has a mild-temperate climate, UVR exposure—both recreational and occupational—may further promote the development of skin malignancies, particularly in chronically photo-exposed areas.

Considering only our patients diagnosed with skin cancer, PLWH were significantly younger than HUPs (average ages of 53.7 years versus 68.1 years), highlighting the importance of not overlooking skin cancer screening and starting it earlier in high-risk groups like the immunosuppressed.

Several pathogenic mechanisms have been proposed to explain the risk of cutaneous malignancies in PLWH, beyond those already established for the general population. PLWH experience a chronic immunosuppressed state that may not be fully reflected by the CD4+ T count or improved by cART. They undergo ongoing antigenic stimulation by HIV, leading to a state of chronic inflammation and cytokine dysregulation that can contribute to the development of lymphomas and other tumors [29,44]. Additionally, the potentially oncogenic HIV proteins tat and nef, which disrupt MHC signaling and chemokine production [49], the high incidence of HPV infection and its possible role in the pathogenesis of SCC [23,50,51], and elevated interleukin levels that promote keratinocyte and melanocyte proliferation could all serve as cofactors in the onset of skin cancers [44].

Notably, in our study, we identified two rare skin cancers (BSC and DFSP) in PLWH that have not been reported in other similar population studies [30,31,43,44,45,46] or reviews/meta-analyses on this subject [29,49,52]. In fact, only a single case report from 25 years ago described the occurrence of DFSP in two patients with HIV infection [53].

## 5. Conclusions

In conclusion, our study shows that PLWH develop skin cancers and precancerous skin conditions at rates similar to those of HUPs. This comparable detection rate between cases and controls might seem unexpected. However, it is important to note that PLWH in our study were specifically asked to undergo a skin cancer screening, and before this invitation, they were unaware of the potential presence of precancerous or cancerous skin lesions. Therefore, it is advisable to increase awareness among healthcare workers who treat HIV and among PLWH about skin cancers to prevent late diagnoses and serious outcomes.

Notably, PLWH develop skin cancers at a younger age and in greater numbers compared to HUPs. Additionally, they may develop uncommon skin cancers such as BSC and DFSP, which often require a multidisciplinary approach (especially for DFSP) at an expert center.

The increased risk of multiple and rare skin cancers in PLWH needs to be properly recognized by both providers and patients. PLWH, especially those with a known history of extensive UVR exposure (both natural and artificial), should receive enhanced medical care along with sun-protective behavioral education. In line with other authors [47], we recommend a baseline dermatology evaluation for all PLWH to assess skin cancer risk, considering personal history of UVR exposure and sunburns, family history of melanoma, skin phototype, and HIV management history. Individuals considered at low skin cancer risk could be managed with thorough patient education and follow-up visits. Those at high risk of cutaneous malignancies should have annual dermatologic exams for skin cancer screening by a trained clinician [47] and should be managed in tertiary care centers. A multidisciplinary team—including infectious disease specialists, dermatologists, plastic surgeons, pathologists, and oncologists—is essential for the best management of these patients [33,54].

## Figures and Tables

**Table 1:** Demographic and clinical features of the studied patients.

Patient’s Features	People Living with HIV (%)	HIV Negative Subjects (%)	*p* Value
Overall	91	91	
Sex			**0.000061**
Male	71 (78.8%)	45 (49.4%)	
Female	20 (21.2%)	46 (50.6%)	
Mean age (range, standard deviation)	52.3 (30–80, ±12.0)	49.9 (19–84, ±17.8)	
Age			0.099946
≤50	34 (37.3%)	45 (49.4%)	
≥50	57 (62.7%)	46 (50.6%)	
Phototype			
II	46 (50.6%)	41 (45.1%)	0.458108
III	39 (42.9%)	49 (53.8%)	0.137993
IV	4 (4.3%)	1 (1.1%)	0.053949
V-VI	2 (2.2%)	0 (0%)	
Melanoma family history			0.096795
yes	1 (1.1%)	5 (5.4%)	
no	90 (98.9%)	86 (94.6%)	
Photodamage			0.656423
yes	45 (49.4%)	48 (52.7%)	
no	46 (50.6%)	43 (47.3%)	
Photosensitizing drugs >5 years			0.850814
yes	17 (18.7%)	18 (19.8%)	
no	74 (81.3%)	73 (80.2%)	
Immunosuppressive drugs			0.173685
yes	1 (1.1%)	4 (4.4%)	
no	90 (98.9%)	87 (95.6%)	
Counting nevi			0.671814
<50	77 (84.6%)	79 (86.8%)	
>50	14 (15.4%)	12 (13.2%)	
Antiretroviral therapy			
yes	89 (97.8%)		
no	2 (2.2%)		

**Table 2:** Detection rate of precancerous skin lesions and of skin cancers in cases and controls (primary aim of the study).

	People Living with HIV (%)	HIV Negative Subjects (%)	*p* Value
Detection of precancerous skin lesions (n° of patients)			0.823468
yes	11 (12%)	12 (13.2%)	
no	80 (88%)	79 (86.8%)	
Detection of skin cancers (n° of patients)			0.78751
yes	7 (7.6%)	8 (8.7%)	
no	84 (92.4%)	83 (91.3%)	

**Table 3:** Characteristics of subjects with skin cancers: clinical and histological features of cutaneous malignancies in PLWH and HUPs.

People Living with HIV (PLWH) with Skin Cancers					
Patient N°	Sex	Age	N° of Skin Cancers Histologically Confirmed	Type of Skin Cancer/Cancers	Site of Skin Cancers
1	M	48	1	BCC	face
2	M	40	1	DFSP	trunk
3	M	75	4	2 MM, 1 BCC, 1 BSC	2 trunk, neck, face
4	M	54	1	KS	head
5	M	62	2	2 SCC	2 trunk
6	M	34	1	KS	trunk
7	M	63	1	BCC	neck
Total **N°** of cancers		53.7	11	3 BCC, 2 MM, 2 SCC, 2 KS, 1 BSC, 1 DPFP	6 trunk, 5 head-neck region
**HIV Uninfected Subjects (HUPs) with Skin Cancers**					
**Patient N°**	**Sex**	**Age**	**N° of Skin Cancers Histologically Confirmed**	**Type of Skin Cancer/Cancers**	**Site of Skin Cancers**
1	M	66	1	BCC	lower limb
2	M	82	1	BCC	upper limb
3	M	76	2	2 BCC	head
4	M	65	1	MM	trunk
5	M	42	1	MM	trunk
6	M	82	1	BCC	trunk
7	M	48	1	BCC	trunk
8	M	84	1	KS	lower limb
Total **N°** of cancers		68.1	9	6 BCC, 2 MM, 1 KS	4 trunk, 3 limbs, 1 head

## Data Availability

Data are available on reasonable request from the corresponding author.

## Abbreviations

The following abbreviations are used in this manuscript:

HIV	Human immunodeficiency virus
PLWH	People living with HIV
HUPs	HIV-uninfected people
BCC	Basal cell carcinoma
SCC	Squamous cell carcinoma
BSC	Baso-squamous carcinoma
DFSP	Dermatofibrosarcoma protuberans
AK	Actinic keratosis
